# The impact of placental pathology discordance in multiple gestation pregnancies on bronchopulmonary dysplasia-associated pulmonary hypertension

**DOI:** 10.1177/2045894020910674

**Published:** 2020-03-09

**Authors:** Andrew Franklin, Sushmita Yallapragada, Robert Birkett, William Grobman, Linda M. Ernst, Karen Mestan

**Affiliations:** 1 Department of Pediatrics, NorthShore University HealthSystem, Evanston, IL, USA; 2 Department of Pediatrics, University of Texas Southwestern, Dallas, TX, USA; 3 Department of Pediatrics, Northwestern University Feinberg School of Medicine, Chicago, IL, USA; 4 Department of Obstetrics and Gynecology, Northwestern University Feinberg School of Medicine, Chicago, IL, USA; 5 Department of Pathology and Laboratory Medicine, NorthShore University HealthSystem, Evanston, IL, USA

**Keywords:** multiple gestation, bronchopulmonary dysplasia-associated pulmonary hypertension (BPD-PH), maternal vascular malperfusion (MVM), placental pathology

## Abstract

Bronchopulmonary dysplasia-associated pulmonary hypertension (BPD-PH) may either be concordant or discordant between multiple gestation births. Abnormal placental development, particularly maternal vascular malperfusion, may account for discordance in BPD-PH through fetal programming mechanisms. Maternal vascular malperfusion is a placental histologic lesion associated with intrauterine growth restriction and BPD-PH. We conducted a retrospective longitudinal cohort study of infants born <29 weeks gestation with available placental histology at Prentice Women's Hospital in Chicago from 2005–2012. The primary outcome was discordant BPD-PH associated with placental maternal vascular malperfusion. We secondarily assessed whether the risk of BPD-PH and placental lesions was different among infants of multiple (compared to singleton) gestations. The cohort consisted of 135 multiple gestation infants and 355 singletons. In a separate cohort of 39 singletons and 35 multiples, associations between 12 cytokines and angiogenic growth factors in cord blood plasma for biomarker discordance, maternal vascular malperfusion, and bronchopulmonary dysplasia were explored. Among multiples, discordant maternal vascular malperfusion was not associated with BPD-PH (OR = 1.9 (0.52, 6.9); *p* = 0.33) in infants exposed to placental maternal vascular malperfusion. However, singleton infants were more likely to develop BPD-PH (compared to multiples) after adjusting for mode of delivery, chorioamnionitis, chronic hypertension, placental abruption, small-for-gestational age birth weight, and gestational age (aOR = 2.7 (1.2, 5.8); *p* = 0.038). Singletons were more likely to be small-for-gestational age (11% vs 4%, *p* = 0.025) and have placental lesions compared to their multiple-gestation counterparts (96% vs 81%, *p* < 0.001), principally severe maternal vascular malperfusion (17% vs 4%, *p* < 0.001) and chronic inflammation (32% vs 11%, *p* < 0.001). Increased risk of BPD-PH in singleton pregnancies <29 weeks gestation compared to multiples may be related to increased frequency of these histologic lesions. Placental pathology in singleton and multiple gestation pregnancies may serve as an early biomarker to predict BPD-PH.

## Introduction

Bronchopulmonary dysplasia (BPD) is the most common chronic lung disease of infancy, with up to 40% of BPD cases complicated by pulmonary hypertension (PH).^1^ When BPD is accompanied by PH (BPD-PH), the risk for death is four-fold greater than when there is BPD alone.^2^ Currently, there are no reliable biomarkers to predict which infants will develop BPD (with or without PH).

Placental histopathology may provide insight as increasing evidence suggests that the process leading to BPD begins before birth with the placenta playing a key role in regulating fetal and neonatal angiogenesis.^3,4^ Thus, fetal programming in the context of an abnormally functioning placenta may predispose an infant to develop BPD-PH postnatally, particularly when maternal vascular malperfusion (MVM) is present.^5–7^ MVM is a placental histologic lesion that often indicates abnormal trophoblast implantation, with subsequent maldevelopment of maternal spiral arteries that give rise to compromised placental–fetal circulation. As such, MVM is commonly present in pregnancies complicated by maternal preeclampsia, intrauterine growth restriction, and stillbirth.^8–10^ Malperfusion of the placental bed, due to a constellation of villous and vascular pathologies, may have direct or indirect impact on the developing pulmonary vasculature.

The ability to discern the associations between MVM and BPD-PH may be enhanced by studying multiple gestation births and their neonatal pulmonary outcomes. Women with twin/triplet pregnancies have an increased frequency of preterm delivery compared to those with singletons. Studies have suggested that preterm birth among women with twins may have different etiologies than preterm birth among women with singletons. The difference in these etiologies is underscored by the higher rates of placental pathologic lesions, including inflammatory and vascular lesions, in singletons.^11–14^ However, to our knowledge, no cohort that includes a large proportion of multiple gestation infants born <29 weeks of gestation has previously evaluated the association between placental histopathology and premature infant outcomes, specifically BPD-PH. We hypothesize that in multiple gestation births, MVM will be associated with the development of BPD-PH, and that MVM discordance will predict BPD-PH discordance. We further hypothesize that in pregnancies less than 29 weeks gestation, placentas from multiple gestation pregnancies will have increased frequency of pathologic lesions, particularly MVM.

## Methods

### Study design and patient sample

We conducted a retrospective cohort analysis of all premature infants born at Northwestern Prentice Women's Hospital in Chicago, IL, USA from 2005–2012. From that population, we included all live-born infants delivering at less than 29 weeks gestational age (GA) at birth with available placental histology for singletons or complete sets of multiples. Placental gross and histopathology is routinely completed for all births less than or equal to 34 weeks of gestation at Prentice, and placental slides and tissue blocks are routinely archived in the Department of Pathology. Small-for-gestational-age (SGA) birth was defined as birth weight-for-GA less than the 10th percentile according to Fenton growth curves.^15^ BPD severity was defined according to NIH consensus criteria of supplemental oxygen use at 36 weeks corrected GA.^16^ Mild BPD was defined as an oxygen requirement at 28 days and breathing room air at 36 weeks corrected GA; moderate BPD was defined as need for <30% oxygen at 36 weeks corrected GA; and severe BPD was defined as ≥30% oxygen or positive pressure ventilation at 36 weeks corrected GA. BPD-PH was defined by echocardiography, according to previously published criteria^5,7^; all premature infants born at <29 weeks received routine echocardiogram screening at one month and 36 weeks corrected GA if they remained on oxygen at these two time points. Intraventricular hemorrhage (IVH) was based on head ultrasound at day of life 7 and 30, with severe IVH defined as grade 3 or 4 based on radiologic criteria by Volpe.^17^ This study was approved by the Institutional Review Board of Northwestern University.

### Perinatal and placental covariates

Maternal and infant clinical data were extracted using standardized abstraction methods performed by Northwestern's Enterprise Data Warehouse. This information included antepartum, intrapartum, and pregnancy outcome data. Maternal hypertensive disorders of pregnancy were defined according to the American College of Obstetrics and Gynecology criteria.^18^ Placental weight and histology were recorded based on standardized gross and histopathologic placental examination that was confirmed by a single perinatal pathologist (L.M.E.) masked to neonatal outcomes. Placental weight <10th percentile was defined as SGA using published nomograms.^19^ Placental histology included evaluation for presence of lesions along four major histopathologic domains: acute inflammation, chronic inflammation, fetal vascular malperfusion, and MVM. Standardized methods for placental pathology reporting and analysis are based upon guidelines from the Amsterdam Placental Workshop Group as previously described for this cohort.^5^ Severe MVM was defined as the presence of both villous and vascular lesions with an accompanying SGA placenta. Villous sublesions of MVM included villous infarcts, increased syncytial knots, villous agglutination, increased intervillous fibrin, and distal villous hypoplasia/small terminal villi. Vascular sublesions of MVM included fibrinoid necrosis/acute atherosis, persistent muscularization of basal plate arterioles, mural hypertrophy of the membrane arterioles, and decidual vascular thrombosis.

### Cord blood biomarker levels and discordance among multiples

We analyzed existent biomarker data from a sample of 74 preterm births enrolled prospectively at the same institution from 2014–2016. The methods for cord blood collection and simultaneous measurement of 12 analytes in archived plasma via multiplex immunoassay have been previously published.^20^ Twins and triplets were identified from the sample and analyzed as matched pairs for percent biomarker discordance, which was calculated similar to birth weight discordance: (absolute difference between sibling biomarker levels divided by the higher level, multiplied by 100). Biomarker discordance (yes/no) was defined as >25% discordance between siblings.

### Statistical analysis

The primary outcome was, among women with multiple gestations, discordant BPD-PH associated with placental MVM. Secondary analyses included the association of multiple gestation (compared to singleton gestation) with neonatal outcomes including BPD, BPD-PH, necrotizing enterocolitis, sepsis, patent ductus arteriosus (PDA), IVH, periventricular leukomalacia, retinopathy of prematurity (ROP), and death. We conducted analyses using both BPD-PH and BPD-PH with death as outcomes. Chi square or student t-tests were used for comparison between singleton and multiple gestations for categorical and continuous data, respectively. Cord blood biomarker data were analyzed using Wilcoxon rank sum tests. Multivariable logistic regression using a generalized estimating equation that accounted for non-independence of observations of infants from the same pregnancy was used to estimate odds ratios (ORs) of neonatal outcomes adjusting for significant covariate differences in baseline characteristics.

## Results

### Baseline characteristics

A total of 490 infants, of whom 135 were from multiple gestations (57 twin gestations and 7 triplet gestations for a total of 64 multiple gestation births) and 355 were from singleton gestation births, were included in this study. The baseline characteristics of the study population, stratified by plurality of gestation, are presented in Table 1. There were no differences between groups in GA, birth weight, sex, and APGAR scores at 1 and 5 min. Women who delivered multiples (twins or triplets) were older, more likely to be white, have preterm labor as a cause of preterm birth, premature rupture of membranes, and have had a cesarean delivery. Women giving birth to singletons more frequently had preeclampsia, hypertensive disorders of pregnancy, chorioamnionitis, oligohydramnios, placental abruption, cesarean section for non-reassuring fetal heart tones, and SGA births. Of the 112 infants from twin gestations, 14 were from monochorionic/diamniotic gestations and 98 were from dichorionic/diamniotic gestations. Among the 21 infants from triplet gestations, 18 were from trichorionic/triamniotic gestations and three were from dichorionic/triamniotic gestations.

**Table 1.** Baseline characteristics of cohort.

	Singleton (*n* = 355)	Multiple (*n* = 135)	*p* Values
Infant characteristics			
Gestational age (weeks)	26.5 (1.6)	26.6 (1.6)	NS
Birth weight (grams)	894 (255)	921 (242)	NS
Small for gestational age (%)	39 (11)	6 (4)	0.025
APGAR 1 min	4 (2.3)	4 (2.2)	NS
APGAR 5 min	6.6 (1.9)	6.7 (1.9)	NS
Maternal characteristics			
Maternal age (years)	29 (6.6)	33.1 (6.4)	<0.001
Male sex (%)	209 (59)	77 (57)	NS
Maternal race (%)			<0.001
Black	126 (36)	19 (14)	
White	93 (26)	87 (64)	
Asian	16 (5)	5 (4)	
Hispanic	42 (12)	8 (6)	
Other	19 (5)	4 (3)	
Unknown	59 (16)	12 (9)	
Preterm labor (%)	242 (68)	120 (89)	<0.001
Premature rupture of membranes (%)	152 (43)	77 (57)	0.017
Prolonged rupture of membranes (%)	92 (26)	23 (17)	NS
Artificial rupture of membranes (%)	164 (46)	57 (42)	NS
Cesarean section (%)	201 (57)	95 (70)	0.019
Antenatal steroids (%)	275 (78)	96 (71)	NS
Preeclampsia (%)	81 (23)	5 (4)	<0.001
Eclampsia (%)	2 (0.5)	0	NS
HELLP (%)	18 (5)	1 (1)	0.027
Pregnancy-induced hypertension (%)	10 (3)	1 (1)	NS
Chronic hypertension (%)	27 (8)	0	0.001
Chorioamnionitis (%)	64 (18)	11 (8)	0.007
Gestational diabetes (%)	18 (5)	4 (3)	NS
Oligohydramnios (%)	26 (7)	0	0.001
Placental abruption (%)	64 (18)	12 (9)	0.013
Non-reassuring fetal heart tones (%)	83 (23)	14 (10)	0.004

### BPD-PH by MVM

Among infants from multiple gestation pregnancies, discordant BPD-PH was present in 9 of the 64 (14%) multiple gestation sibling pairs/trios. Seven of the 64 (11%) had discordant MVM with only one sibling grouping having both discordant MVM and discordant BPD-PH, but not both MVM and BPD-PH. In multivariate analysis, MVM was not significantly associated with BPD-PH (OR = 1.9 (0.52, 6.9); *p* = 0.33). In the entire cohort, including both singletons and multiple gestation infants, both any MVM (OR = 1.9 (1.1, 3.2), *p* = 0.02) and severe MVM (OR = 3.2 (1.8, 6.0), *p* <0.001) were associated with development of BPD-PH.

### Neonatal outcomes in singleton vs multiple gestation

BPD-PH was significantly more frequent in infants from singletons compared to those from multiple gestations (Table 2; 16 vs 7%; OR = 2.7 (1.3, 5.6), *p* = 0.008). Singleton infants were also more likely to have sepsis and require laser surgery for ROP. However, there were no differences between these two groups in the frequency of any BPD, necrotizing enterocolitis, PDA, IVH, periventricular leukomalacia, ROP, or death. Singleton infants remained more likely to develop BPD-PH compared to multiple gestation neonates after adjusting for mode of delivery, chorioamnionitis, chronic hypertension, placental abruption, SGA status, and gestational age (adjusted OR = 2.7 (1.2, 5.8); *p* = 0.038).

**Table 2.** Neonatal outcomes by multiple gestation.

	Singleton (*n* = 355)	Multiple (*n* = 135)	*p* values
Any BPD	229 (65)	77 (57)	NS
Mild BPD	85 (24)	38 (26)	NS
Moderate BPD	72 (20)	23 (17)	NS
Severe BPD	63 (18)	17 (13)	NS
Severe BPD or death	80 (23)	27 (20)	NS
Pulmonary hypertension	57 (16)	9 (7)	0.007
Pulmonary hypertension or death	76 (21)	19 (14)	0.07
Necrotizing enterocolitis			NS
Confirmed pneumatosis	78 (22)	22 (16)	
Presumed NEC	26 (7)	10 (7)	
Sepsis	101 (28)	25 (19)	0.03
Patent ductus arteriosus	165 (46)	65 (48)	NS
PDA ligation	56 (16)	16 (12)	NS
Any IVH	157 (44)	51 (38)	NS
Severe IVH	53 (15)	22 (16)	NS
Periventricular leukomalacia	15 (4)	7 (5)	NS
Retinopathy of prematurity	164 (46)	57 (42)	NS
ROP stage 2+	86 (24)	27 (20)	NS
ROP laser	39 (11)	7 (5)	0.047
Disposition			
Home	263 (74)	116 (86)	0.005
Transfer	70 (20)	9 (7)	<0.001
Death	22 (6)	10 (7)	NS

BPD: bronchopulmonary dysplasia; NEC: necrotizing enterocolitis; PDA: patent ductus arteriosus; IVH: intraventricular hemorrhage; ROP: retinopathy of prematurity.

### Placental histopathologic domains in singleton vs multiple gestation

**Fig. 1.**
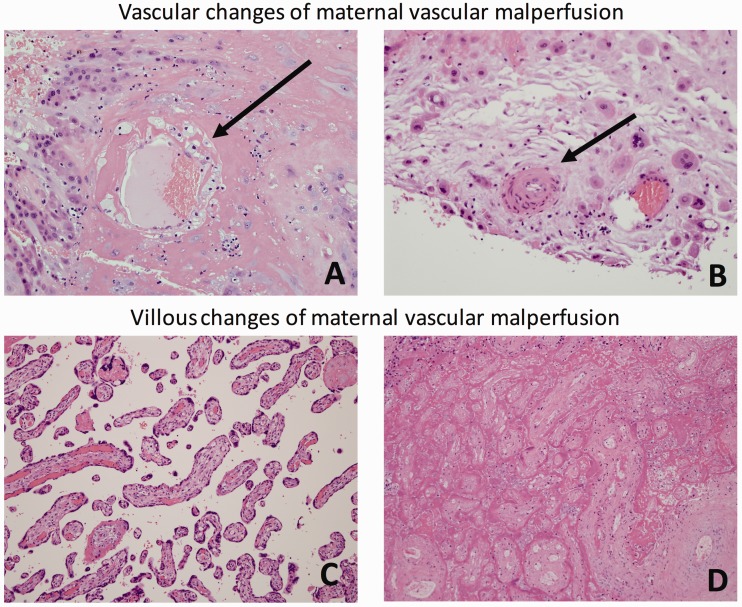
Histologic lesions of maternal vascular malperfusion. (a) Decidual vessel showing acute atherosis characterized by fibrinoid necrosis of the vascular wall and numerous foamy macrophages (arrow) within the wall. (b) Basal decidual vessel with persistent muscularization characterized by a thick coat of smooth muscle (arrow). (c) Chorionic villi showing distal villous hypoplasia characterized by long, slender villi with reduced villous branching. (d) Villous infarction characterized by collapse of the intervillous space and coagulative necrosis of the villous tissue.

Singletons were more likely to have a placenta with at least one histopathologic lesion in any domain (acute inflammation, chronic inflammation, fetal vascular pathology, or MVM) compared to multiples (Table 3; 96% of singletons vs 81% of multiples, *p* <0.001). Singletons were more likely to have placental lesions of acute inflammation including both high-stage maternal and fetal inflammation, funisitis, peripheral funisitis, and villous edema compared to multiple gestations. Singleton placentas were more likely to have chronic inflammatory lesions, specifically, chronic deciduitis with plasma cells. Singletons were also found to have increased incidence severe MVM, particularly vascular sublesions of MVM including fibrinoid necrosis/acute atherosis, muscularization of the basal plate arterioles, mural hypertrophy of the membrane arterioles, and basal/decidual vascular thrombosis. Villous sublesions of villous infarcts and distal villous hypoplasia/small terminal villi were also more common in singletons compared with multiple gestation placentas (see Fig. 1). There were no differences in the frequency of fetal vascular pathology between groups.

**Table 3.** Placental histologic domains by multiple gestation.

	Singleton (*n* = 355)	Multiple (*n* = 135)	*p* Values
Placental weight (grams)	227 (79)	313 (189)	<0.001
Small for gestational age placenta	97 (27)	48 (36)	NS
Bilobed placenta	0	2 (1)	0.022
Fibroid	3 (1)	0	NS
Any placental lesion	341 (96)	109 (81)	<0.001
Acute inflammation			
Acute inflammation	216 (61)	72 (53)	NS
Maternal inflammation	215 (61)	70 (52)	NS
Maternal high-stage inflammation	161 (45)	40 (30)	0.002
Fetal inflammation	168 (47)	43 (32)	0.002
Fetal high-stage inflammation	129 (36)	23 (17)	<0.001
Funisitis	126 (35)	22 (16)	<0.001
Peripheral funisitis	36 (10)	4 (3)	0.01
Villous edema	128 (36)	29 (21)	0.002
Chronic inflammation			
Chronic inflammation	114 (32)	15 (11)	<0.001
Chronic villitis	8 (2)	0	NS
Chronic basal villitis	9 (3)	0	NS
Chronic deciduitis with plasma cells	70 (20)	11 (8)	0.002
Chronic chorion and amnion inflammation	30 (8)	2 (2)	NS
Chronic decidual perivasculitis	4 (1)	0	NS
Chronic intervillositis	7 (2)	0	NS
Fetal vascular malperfusion			
Fetal vascular pathology	66 (19)	26 (19)	NS
Thrombi	35 (10)	18(13)	NS
Thrombi chorionic vessels	28 (8)	11 (8)	NS
Thrombi stem villous vessels	10 (3)	7 (5)	NS
Thrombi umbilical vessels	4 (1)	0	NS
Avascular villi	37 (10)	18 (13)	NS
High-grade fetal vascular malperfusion	11 (3)	3 (2)	NS
Maternal vascular malperfusion			
MVM any	177 (50)	55 (41)	0.07
MVM mild	53 (15)	24 (18)	NS
MVM moderate	64 (18)	25 (19)	NS
MVM severe	60 (17)	6 (4)	<0.001
MVM vascular sublesions			
Any maternal vessel pathology	112 (32)	14 (10)	<0.001
Fibrinoid necrosis/acute atherosis	55 (17)	2 (1)	<0.001
Muscularization of the basal plate arterioles	73 (21)	4 (3)	<0.001
Mural hypertrophy of the membrane arterioles	47 (13)	6 (4)	0.005
Basal/decidual vascular thrombosis	20 (6)	2 (1)	0.047
MVM villous sublesions			
Any maternal villous changes	162 (46)	52 (39)	NS
Villous infarcts	52 (15)	5 (4)	0.001
Increased syncytial knots	150 (42)	46 (34)	NS
Villous agglutination	21 (6)	5 (4)	NS
Increased perivillous fibrin	27 (8)	13 (9)	NS
Distal villous hypoplasia/small terminal villi	138 (39)	39 (29)	0.04
Other placental pathology			
Histologic evidence of abruption	69 (19)	19 (14)	NS
Remote membrane hemorrhage with hemosiderin	31 (9)	5 (4)	0.06
Basal plate with hemosiderin	21 (6)	2 (1)	0.04
Chorioamnionic hemosiderosis	18 (5)	7 (5)	NS
Massive perivillous fibrin deposition	3 (1)	0	NS
Subchorionic/intervillous hemorrhage/thrombosis	24 (7)	8 (6)	NS
Amnion nodosum	8 (2)	2 (1)	NS
Umbilical cord abnormality			
Any cord abnormality	49 (14)	22 (16)	NS
Hypercoiling	20 (6)	0	0.005
Velamentous insertion	7 (2)	15 (11)	<0.001
Marginal insertion	17 (5)	10 (7)	NS
Pseudoknot	2 (1)	0	NS
Two vessel cord	2 (1)	1 (1)	NS

MVM: maternal vascular malperfusion.

Notes: Numbers represent means (SD) or number (%). Statistics calculated using Chi squared or student t-tests where appropriate with significance defined as *p* < 0.05. Any placental lesion includes acute inflammation, chronic inflammation, fetal vascular malperfusion, and maternal vascular malperfusion.

### Cord blood biomarker levels and discordance among multiples

In the second sample of 74 preterm births (mean GA 30.8 ± 2.1; mean birth weight = 1494.0 ± 373.8), 39 singletons and 35 multiples were identified. All had complete cord blood biomarker data as well as corresponding clinical outcomes and placental histology data. Median biomarker levels of the 12 analytes according to singleton and multiple gestational status are shown in Table 4. Among the multiples, 13 completely matched pairs of twins and one set of triplets were identified. The remaining were unmatched multiples. Biomarker discordance was highly variable among the 12 analytes, with median values ranging from 17% (endoglin) to 81% (epidermal growth factor (EGF)) (see Table 4). Only one set of twins were monochorionic/diamniotic the remaining were di/di. Upon restriction to di/di twins, the biomarker discordance patterns did not change, with the exception of two biomarkers (EGF and angiopoietin-2) reaching the significance threshold (*P* <0.05) for MVM and villous changes. There were seven cases of PH disease at 36 weeks corrected age, but only one patient was a multiple. This twin with PH had MVM on placental histology, while the sibling had neither MVM nor PH disease. Unfortunately, cord blood was not obtainable from the non-affected twin, so biomarker discordance could not be determined.

**Table 4.** Cord blood biomarker levels and percent discordance among siblings in multiple gestation births, according to placental MVM and BPD.

	EGF	ANG2	G-CSF	BMP9	ENG	ET1	IL-8	HGF	HBEGF	PLGF	FGF2	VEGF-A
Cord blood plasma pg/mL, median (IQR)												
Singleton, *n* = 39	13 (3, 66)	4664 (2887, 9876)	84 (34, 1115)	261 (207, 483)	1151 (759, 2074)	9 (4, 13)	16 (4, 86)	396 (145, 806)	35 (13, 86)	3 (1, 8)	49 (30, 105)	13 (13, 88)
Multiple, *n* = 35	8 (3, 28)	3867 (2512, 9026)	65 (23, 136)	241 (153, 401)	1042 (702, 2109)	4 (3, 12)	9 (4, 20)	243 (139, 676)	23 (13, 83)	3 (1, 7)	47 (24, 134)	13 (13, 23)
% discordance, median (IQR)												
All, *n* = 29	81 (35, 89)	19 (5, 40)	35 (25, 71)	29 (22, 54)	17 (7, 61)	33 (4, 66)	58 (40, 71)	29 (24, 62)	38 (29, 85)	45 (25, 59)	57 (39, 73)	23 (0, 52)
Any MVM												
No, *n* = 7	89 (67, 98)	29 (19, 84)	31 (29, 74)	22 (22, 54)	17 (5, 76)	42 (8, 93)	63 (35, 69)	29 (5, 77)	66 (30, 86)	45 (44, 86)	54 (44, 91)	52 (23, 83)
Yes, *n* = 22	81 (20, 88)	14 (5, 24)^b^	35 (24, 53)	31 (15, 45)	11 (7, 26)	25 (0, 59)	56 (40, 77)	35 (24, 58)	38 (21, 66)	46 (11, 57)	60 (37, 73)	0 (0, 46)^a^,^b^
Vessel path												
No, *n* = 27	87 (35, 89)	19 (5, 40)	35 (25, 71)	28 (22, 54)	17 (7, 61)	33 (3, 66)	58 (40, 71	29 (24, 62)	38 (29, 85)	45 (11, 59)	57 (39, 73)	0 (0, 0)
Yes, *n* = 2	36 (0, 71)	21 (1, 40)	30 (25, 35)	20 (6, 33)	15 (4, 26)	31 (3, 59)	) 49 (7, 92)	28 (16, 41)	32 (21, 43)	38 (25, 51)	QNS	51 (37, 66)
Villous changes												
No. *n* = 8	80 (69, 98)	29 (10, 80)	31 (27, 73)	22 (14, 54)	17 (4, 74)	42 (6, 88)	60 (21, 66)	23 (11, 77)	54 (37, 85)	45 (34, 83)	54 (44, 74)	59 (38, 74)
Yes, *n* = 21	81 (20, 88)	14 (5, 24)^b^	35 (24, 53)	33 (22, 45)	11 (8, 26)	25 (0, 62)	58 (40, 77)	41 (25, 58)	38 (21, 66)	46 (11, 57)	64 (35, 73)	0 (0, 37)^a^,^b^
BPD												
No, *n* = 24	81 (35, 89)	24 (10, 40)	35 (29, 73)	29 (22, 45)	14 (6, 26)	42 (8, 66)	60 (40, 70)	29 (24, 58)	38 (25, 66)	5 (25, 59)	49 (35, 64)	37 (0, 57)
Yes, *n* = 5	88 (20, 88)	13 (4, 13)	24 (23, 52)	71 (5, 91)	61 (7, 80)	0 (0, 66)	55 (5, 77)	62 (25, 62)	89 (38, 89)	46 (0, 46)	73 (72, 96)^a^,^b^	0 (0, 0)^a^,^b^

EGF: epidermal growth factor; ANG2: angiopoietin-2; G-CSF: granulocyte-colony stimulating factor; BMP9: bone morphogenic protein-9; ENG: endoglin; ET1: endothelin-1; IL-8: interleukin-8; HGF: hepatic growth factor; HBEGF: heparin binding EGF-like growth factor; PLGF: placental growth factor; FGF2: fibroblast growth factor-2; VEGF-A: vascular endothelial growth factor-A; QNS: quantity not sufficient for analysis; MVM: maternal vascular malperfusion.

Notes: % discordance calculated using biomarker levels between siblings (absolute difference between levels divided by the higher level, multiplied by 100).

BPD defined as moderate–severe disease (NIH consensus criteria) or death.

a *p* < 0.05; data presented as median (IQR) and compared using Wilcoxon rank-sum tests.

b *p* < 0.05; after further restriction to di/di twins (monochorionic/diamniotic twins removed).

## Discussion

To our knowledge, this is the first study to investigate associations of placental pathology on BPD-PH in a large cohort of singleton and multiple gestation preterm infants. We found that discordant BPD-PH in multiples was not associated with discordant placental MVM in this cohort. However, we found a 2.7-fold increased odds of BPD-PH in singletons compared to multiple gestation infants. Furthermore, there was an increased frequency of SGA infants, placental lesions (particularly MVM, acute inflammation, and chronic inflammation), sepsis, and ROP surgery in singletons compared to multiple gestation placentas.

This study did not support our hypothesis that discordant MVM was associated with discordant BPD-PH in multiple gestation infants. This may be because our sample size of multiple gestation dyads and triads was not sufficiently large and had a relatively low incidence of MVM and BPD-PH discordance among the groups. It also may be related to our finding of fewer placental pathologic lesions in multiple gestation pregnancies. However, in the overall cohort, we show a three-fold increase in odds of development of BPD-PH with severe MVM, which supports previous studies showing this association.^5–7^

This study shows a novel finding of increased risk of developing BPD-PH in singletons compared to multiple gestation infants. There were more placental histopathologic lesions from multiple domains in singleton placentas. Specifically, we found that MVM was more frequent in singleton placentas as compared with placentas from multiple gestations. Perhaps this may reflect the different reasons that multiple gestation pregnancies may deliver preterm compared to singletons.^8,21–23^ Compared to previous studies looking at placental pathology in preterm birth, this study was unique in that the cohort was less than 29 weeks gestation, so by inclusion, all infants are born premature. Our data, mirroring previous studies, shows more placental pathologic lesions in singletons compared to multiple gestation preterm placentas.^11–14,24^ The difference in frequency of placental lesions suggests the underlying etiology for preterm birth may be different in singleton and multiple gestations.

In the second sample of 74 births with corresponding cord blood biomarker data, analysis of multiples versus singletons support the findings of the retrospective cohort that MVM discordance and PH disease among multiples is relatively rare. We also found that biomarker discordance in cord blood varies widely between siblings. We speculate that this may be dependent upon the type of cytokine/growth factor, with factors known to regulate angiogenesis (angiopoietin-2, endoglin, and vascular endothelial growth factor-A (VEGF-A)) having apparently lower (although not statistically significant) discordance as compared with inflammatory cytokines (e.g. interleukin-8, granulocyte-colony stimulating factor). Overall, the biomarkers did not vary significantly among singletons and multiples. This is contrary to our findings of placental vascular pathology in the retrospective cohort. Furthermore, we found that discordances of certain angiogenic factors (angiopoietin-2 and VEGF-A) were lower with placental MVM, in particular with villous changes (Table 4). Collectively, these findings might suggest that angiogenic balance among twins may play a role in modifying placental vascular changes in multiple gestation. While there were some signals of increased FGF-2 discordance and decreased VEGF-A discordance with BPD, the sample size was very small and further investigation in a larger sample is warranted.

There are several strengths of this study. To our knowledge, this is the largest study of BPD-PH outcomes among preterm infants that takes into account multiple gestation status and the presence of discordance versus concordance in placental pathology and lung vascular phenotypes. Monochorionic, dichorionic, and trichorionic gestations were included in this study, expanding the sample size and generalizability. This cohort also has comprehensive infant, maternal, and placental data, allowing for investigation of a wide range of covariates of preterm birth, BPD, and PH. Placental histology was performed masked to the study question and BPD-PH outcomes. This study was limited by the small sample of BPD-PH discordant twins; however, we may be the first to report on the incidence of BPD-PH discordance in a population of <29 week infants. Similarly, we report that the occurrence of MVM discordance was infrequent in this preterm population, a finding also not previously reported in the literature. This study was limited by the low number of multiple gestation infants who developed BPD-PH requiring grouping of mono, di, and tri-amniotic and chorionic placentas, which did not allow for assessing the impact of different types of placentation on BPD-PH risk.

To conclude, this study validates our previous report that placental pathologic changes of MVM are associated with the development of BPD-PH.^5^ MVM discordance, however, is not associated with the development of BPD-PH or with BPD-PH discordance. In addition, there are also fewer pathologic abnormalities among placentas from preterm multiple gestations, supporting the concept that the etiologies of preterm birth are dissimilar among women with differing plurality. In larger studies linked to closely associated biomarkers, validation of these findings can elucidate mechanisms of the early fetal origins leading to neonatal pulmonary hypertension.
